# Molecular detection of vector-borne agents in dogs from ten provinces of China

**DOI:** 10.1186/s13071-015-1120-y

**Published:** 2015-10-01

**Authors:** Da Xu, Jilei Zhang, Zhengsheng Shi, Chunlian Song, Xiaofeng Zheng, Yi Zhang, Yongqing Hao, Haiju Dong, Lanjing Wei, Heba S. El-Mahallawy, Patrick Kelly, Wenbin Xiong, Heng Wang, Jianji Li, Xinjun Zhang, Jianhong Gu, Chengming Wang

**Affiliations:** Jiangsu Co-innovation Center for Prevention and Control of Important Animal Infectious Diseases and Zoonoses, Yangzhou University College of Veterinary Medicine, Yangzhou, Jiangsu 225009 P. R. China; China Agricultural University College of Veterinary Medicine, Beijing, 100083 China; Yunnan Agricultural University College of Animal Science & Technology, Kunming, Yunnan 650201 China; Jiangsu Agri-animal Husbandry Vocational College, Taizhou, Jiangsu China; Xinjiang Agricultural University College of Veterinary Medicine, Urumchi, Xinjiang 830052 China; Inner Mongolia Agricultural University College of Veterinary Medicine, Hohhot, Inner Mongolia 010018 China; Henan Agricultural University College of Animal Science and Veterinary Medicine, Zhengzhou, Henan 45002 China; Faculty of Veterinary Medicine, Suez Canal University, Ismailia, 41522 Egypt; Ross University School of Veterinary Medicine, St. Kitts & Nevis, West Indies

**Keywords:** China, Vector-borne agents, FRET-PCR, Dogs

## Abstract

**Background:**

Although many vector-borne agents are potential zoonoses and cause substantial morbidity and mortality in dogs worldwide, there are limited data on these organisms in dogs of China.

**Methods:**

Quantitative PCRs for vector-borne agents were performed to investigate their prevalences in convenience whole blood samples obtained from 1114 dogs from 21 veterinary clinics and a commercial dog breeding facility in ten provinces of China. In addition, the PCRs were performed on 146 *Rhipicephalus sanguineus* senso lato and 37 *Linognathus setosus* collected from dogs in the commercial dog breeding facility.

**Results:**

DNAs of *Babesia gibsoni* and *B. vogeli* (1.2 %), *Ehrlichia canis* (1.3 %), *Hepatozoon canis* (1.8 %) and *Theileria orientalis* (0.1 %) or a closely related organism were detected in the bloods of the dogs studied, and *Babesia vogeli* (3.4 %) and *Ehrlichia canis* (4.1 %) in *R. sanguineus* senso lato. The qPCRs for *Anaplasma* spp., *Dirofilaria immitis* and *Leishmania* spp. were negative for all blood samples, ticks and lice. At least one vector-borne agent was found in dogs from 5 of the 10 provinces investigated in this study. Overall, 4.4 % (49/1117) of the dogs studied were positive for at least one vector-borne agent with the prevalence being highest in the commercial breeding colony (24/97; 24.7 %).

**Conclusions:**

Our study confirms that *B. vogeli*, *B. gibsoni*, *H. canis*, and *E. canis* occur in China. Also, we present evidence that *T. orientalis* or a closely related organism can infect dogs.

## Background

Vector-borne diseases are important causes of morbidity and mortality in dogs and some have also emerged as a significant threat to human health worldwide [1–5]. Records of pet and guard dogs in China can be found dating 8000 thousand years ago and the current dog population is estimated to be between 150 and 200 million [6]. There is very little reliable information on vector-borne agents in dogs in China with only eight reports for the entire country: three using serology (7, 10, 11), one using PCR (13), three using serology and PCR (9, 14, 15), and one using PCR and microscopy (12). Studies with PCR and/or serology methods have indicated the *E. canis* occurs in dogs from Shenzhen, Guangzhou and Hong Kong [7–9] and *D. immitis* in dogs from Beijing, Shanghai, Chongqing, Yunnan, Guangdong and Shenyang provinces/ municipalities [7, 10, 11]. Molecular based studies have shown *D. immitis* in dogs from Liaoning province [12] and leishmaniasis to be common in dogs in Sichuan province [13–15]. Studies on ticks have revealed *A. phagocytophilum* in *Haemaphysalis longicornis* and *Ixodes persulcatus* from Suifenhe on the China-Russia border and *Babesia* spp. in dog ticks from six provinces/municipalities of China [16].

To provide further information on vector-borne agents in dogs in China, we investigated the prevalences of seven organisms (*Babesia*, *Ehrlichia*, *Anaplasma*, *Dirofilaria immitis*, *Theileria*, *Hepatozoon* and *Leishmania*) in blood samples from dogs in 10 provinces of China, and from ticks and lice collected from dogs in one province. Our findings are reported below.

## Methods

### Collection of whole blood and external parasites

**Fig. 1:**
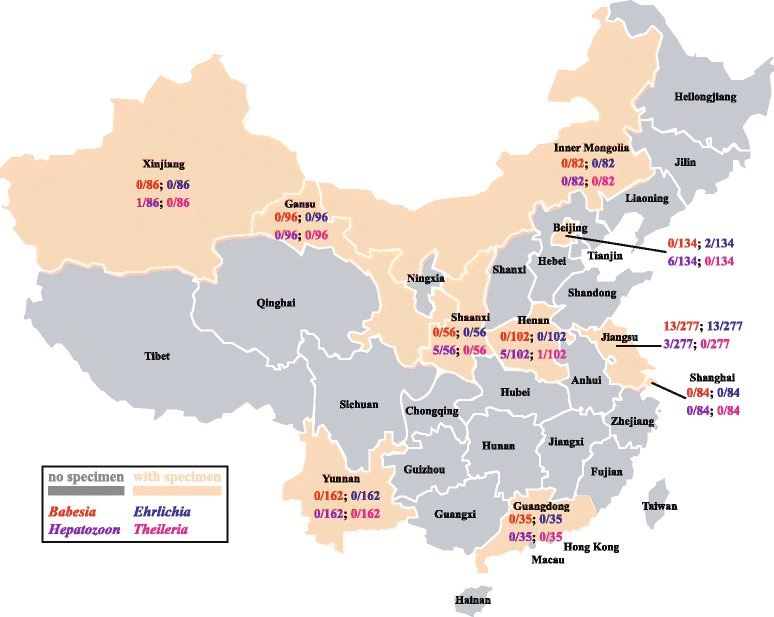
Prevalences of 4 vector-borne agents in 10 provinces of China. Dog blood samples (1114) were collected from ten provinces (in bisque) of China. The prevalences are shown for *Babesia vogeli* (red), *B. gibsoni* (black), *Hepatozoon canis* (green) and *Ehrlichia canis* (blue) in different colors

Between November 2012 and February 2014, convenience whole blood samples were collected in EDTA by veterinarians who volunteered to participate (Table 1, Fig. 1). The dogs sampled in Taixing, Jiangsu province, were apparently healthy animals in a commercial dog breeding facility: convenience samples of ticks and lice were also collected from these dogs. The remaining dogs in the study were those attending 21 local veterinary clinics for routine health checks, vaccinations and for various conditions. Veterinarians were asked to comment on the presence or absence of ectoparasites on the dogs sampled.

**Table 1:** The distribution of studied samples in ten provinces/municipalities

Sample type	Source of samples			
	Province/Municipality	City	Coordinates	Number
Dog blood	Beijing	Beijing	39°N, 116°E	134
	Gansu	Lanzhou	36°N, 103°E	96
	Guangdong	Guangzhou	23°N, 113°E	35
	Henan	Zhengzhou	34°N, 113°E	102
	Inner Mongolia	Huhhot	40°N, 111°E	82
	Jiangsu	Yangzhou	32°N, 119°E	50
		Taixing	32°N, 120°E	97
		Nanjing	32°N, 118°E	130
	Shanghai	Shanghai	31°N, 121°E	84
	Shaanxi	Yangling	34°N, 108°E	56
	Xinjiang	Urumchi	43°N, 87°E	86
	Yunnan	Kunming	25°N, 102°E	162
Ticks	Jiangsu	Taixing	32°N, 120°E	146
Lice	Jiangsu	Taixing	32°N, 120°E	37

Blood samples were frozen at −20 °C before being put on ice (over 2 days) and sent to Yangzhou University College of Veterinary Medicine where they were frozen at −80 °C until thawed at room temperature for DNA extraction. The external parasites collected from the dogs were identified following standard morphological criteria [17] and stored in vials with RNA/DNA Stabilization Reagent for Blood/Bone Marrow (Roche Diagnostics GmbH, Mannheim, Germany) at −80 °C until DNA extraction.

### Ethical approval

The study was approved by the Institutional Animal Care and Use Committee of Yangzhou University, China. Written permission for sampling was obtained from the owners of dogs that participated in the study.

### DNA extraction from whole blood, ticks and lice

Aliquots (200 μL) of whole blood were used for DNA extraction with High Pure PCR Template Preparation Kit (Roche Diagnostics GmbH, Mannheim, Germany) according to the manufacturer’s instructions. The DNAs were eluted with 200 μL Elution Buffer. Ticks and lice were individually homogenized in a shaker (Bertin Technologies, France) with four 3.0 mm ceramic beads for two periods of 15 s (3160 × g with a 15-s break in between). DNAs were extracted from the homogenates with the QIAgen® DNA Mini Kit (Qiagen, Valencia, CA, USA), eluted in 200 μL of 1 × T_10_E_0.1_ buffer, and stored at −80 °C until PCR was performed.

### Quantitative FRET-PCRs

Eight quantitative PCRs, seven for vector-borne agents and one for the mammalian HMBS gene as an endogenous internal control, were performed on a Roche LightCycler 480-II PCR Instrument. The qPCRs were performed as described previously for *Anaplasma* spp. [5], *Babesia* spp. [18], *Dirofilaria immitis* [19], *Ehrlichia* spp. [5], *Hepatozoon* spp. [20], *Leishmania* spp. [21], *Theileria* spp. [22] and the mammalian HMBS gene [23]. The positive PCR products were verified by gel electrophoresis and sequenced using forward and antisense primers (BGI, Shanghai, China).

### Standard PCR

When *T. orientalis* was identified in the *Theileria* qPCR we used a standard PCR amplifying a 591 bp section (including 60-bp primers) of the 18S rRNA gene of *Theileria* spp. [22] to obtain longer sequences for analysis and further verification of identification.

## Results

Information provided by veterinarians at the clinics where the study was performed indicated the vast majority of the dogs we sampled were pets that spent most of their time indoors. It was only very infrequently that the veterinarians noted ectoparasites on such dogs as they had very limited exposure to the outside environment and other animals. Ticks and lice were, however, found on the dogs in the Taixing commercial dog breeding facility which were housed more extensively. These were identified as *Rhipicephalus sanguineus* senso lato (*n* = 146) and *Linognathus setosus* (*n* = 37).

The qPCR for the HMBS gene was positive on all samples with 4.5 × 10^6^ ± 3.9 × 10^5^ copies/ml whole blood.

Overall, 4.4 % (49/1117) of dogs were positive for at least one vector-borne agent and we obtained positive qPCR results for 4 of the 7 organisms we studied; there were only negative results for *Anaplasma* spp., *D. immitis* and *Leishmania* spp. At least one vector-borne agent was identified in 5 of the 10 provinces studied (Fig. 1). The prevalence of vector-borne agents was highest in the dogs from the commercial breeding facility in Taixing (24/97, 24.7 %) where external parasites were observed and collected. Six of the 146 (4.1 %) *R. sanguineus* senso lato collected were positive for *E. canis* but none of the *L. setosus* had evidence of a vector-borne agent (Tables 2 and 3).

**Table 2:** Prevalences of vector-borne agents detected by qPCR in dog bloods, ticks and lice in China

Sample type	Source of samples		Percent (n) PCR positive			
	Province/Municipality	City	*Hepatozoon*	*Ehrlichia*	*Babesia*	*Theileria*
Dog blood	Beijing	Beijing	4.5 % (6/134)	1.5 % (2/134)	0 % (0/134)	0 % (0/134)
	Gansu	Lanzhou	0 % (0/96)	0 % (0/96)	0 % (0/96)	0 % (0/96)
	Guangdong	Guangzhou	0 %, (0/35)	0 % (0/35)	0 % (0/35)	0 % (0/35)
	Henan	Zhengzhou	4.9 % (5/102)	0 % (0/102)	0 % (0/102)	1.0 % (1/102)
	Inner Mongolia	Huhhot	0 % (0/82)	0 % (0/82)	0 % (0/82)	0 % (0/82)
	Jiangsu	Yangzhou	0 % (0/50)	0 % (0/50)	0 % (0/50)	0 % (0/50)
		Taixing	0 % (0/97)	13.4 % (13/97)	11.3 % (11/97)	0 % (0/97)
		Nanjing	2.3 % (3/130)	0 % (0/130)	1.5 % (2/130)	0 % (0/130)
	Shanghai	Shanghai	0 % (0/84)	0 % (0/84)	0 % (0/84)	0 % (0/84)
	Shaanxi	Yangling	8.9 % (5/56)	0 % (0/56)	0 % (0/56)	0 % (0/56)
	Xinjiang	Urumchi	1.2 % (1/86)	0 % (0/86)	0 % (0/86)	0 % (0/86)
	Yunnan	Kunming	0 % (0/162)	0 % (0/162)	0 % (0/162)	0 % (0/162)
			1.8 % (20/1114)	1.3 % (15/1114)	1.2 % (13/1114)	0.1 % (1/1114)
Ticks	Jiangsu	Taixing	0 % (0/146)	4.1 % (6/146)	3.4 % (5/146)	0 % (0/146)
Lice	Jiangsu	Taixing	0 % (0/37)	0 % (0/37)	0 % (0/37)	0 % (0/37)

**Table 3:** Comparison of DNA sequences identified in this study with those having similar gene sequences in GenBank

Isolates identified in this study			Highly similar sequences in GenBank		
Species	GenBank #	Source	GenBank #	Source	Mismatch
*E. canis*	KP719093	13 dogs from Taixing	AF536827	Blood of dog from Kagoshima, Japan	1/158
		2 dogs from Beijing			
	KP719094	6 ticks (*R. sanguineus* senso lato)			
		from Taixing			
*B. vogeli*	KP719088	11 dogs from Taixing	KJ939326	Blood of *Springer Spaniel* from Nanning, Guangxi, China	1/210
	KP719089	5 ticks (*R. sanguineus* senso lato)			
		from Taixing			
*B. gibsoni*	KP719090	2 dogs from Nanjing	LC012808	Blood of dog from Yamaguchi, Japan	0/210
*H. canis*	KP719091	3 dogs from Nanjing	KM115995	Spleen of infected *Vulpes vulpes* from Lower Austria, Gaenserndorf, Austria	0/144
		6 dogs from Beijing			
		5 dogs from Zhengzhou			
		1 dogs from Urumchi			
		5 dogs from Yangling			
*T. orientalis*	N/A	1 dog from Zhengzhou	KM609973	Blood of infected *Bubalus bubalis* from India	0/177
					0/591

The most common organism we identified was *Hepatozoon canis* with 1.8 % (20/1114) of the dogs having PCR evidence of infection (Table 2). The prevalences of infection in Jiangsu (1.1 %; 3/277) and Xinjiang provinces (1.2 %; 1/86) were much lower than those found in Shaanxi (8.9 %; 5/56), Henan (4.9 %; 5/102) and Beijing (4.9 %; 6/134). Genomic sequencing and BLASTN demonstrated that the partial 18S rRNA gene sequences for the *H. canis* we identified and deposited in GenBank database (Gene Accession #: KP719091) were identical to one another, and also to that of a *H. canis* found in the spleen of a red fox in Austria (KM115995) (Table 3).

Overall, we found *Ehrlichia canis* DNA in 1.4 % of the dog blood samples (15/1114) and 4.1 % of the ticks (6/146). Ticks and most of the dogs positive for *E. canis* were from the Taixing commercial dog breeding facility (13/97 dogs, 13.4 %; 6/146 ticks, 4.1 %). Only 2 of the positive dogs were from veterinary clinics (Beijing) where their clinical records showed them to be anemic. The sequences of the *E. canis* we found in the dogs and ticks (KP719093, KP719094) were identical to each other but showed 1 nucleotide mismatch with the sequence of the most closely related *E. canis* which was found in a dog in Japan (AF536827) (Table 3).

Thirteen dogs (13/1114, 1.2 %) and 5 ticks (5/146, 3.4 %) were positive for *Babesia* spp. and sequencing showed two *Babesia* spp. occurred, most commonly *B. vogeli* (11 dogs and all ticks) followed by *B. gibsoni* (2 dogs). The sequences of the *B. vogeli* (KP719088, KP719089) from the dogs and ticks were identical but had 1 mismatch with the sequence of a *B. vogeli* reported in a dog from China (KJ939326). The *B. gibsoni* sequences (KP719090) were identical to each other and also to a *B. gibsoni* in a dog from Japan (LC012808) (Table 3).

A 5-year old, female Chihuahua dog with a perineal hernia seen in a veterinary clinic in Henan province was the only dog found to be positive for *Theileria* spp. The 18S rRNA sequence of the *Theileria* spp. we identified in our qPCR was identical to that of *T. orientalis* Thrissur 1 from India (KM609973) and Japan (XR 696404). Similarly, the sequence of the 591 bp amplicon of the 18S rRNA gene we obtained with a subsequent standard PCR [22] was also identical to that of *T. orientalis* (KM609973, XR_696404).

Multiple infections with vector-borne agents were rare with two dogs and a tick having evidence of infection with *H. canis* and *B. gibsoni*, one dog with *E. canis* and *B. vogeli*, and one tick with *B. vogeli* and *E. canis*.

## Discussion

To date there have only been a few studies on vector-borne agents in Chinese dogs [7–9, 24, 25] but our study has confirmed that *B. vogeli*, *B. gibsoni*, *E. canis, H. canis and T. orientalis* or a closely related organism occur in China. The first three agents are very important pathogens of dogs and veterinarians in China should have an increased awareness of the possibility of infections in their canine patients and appropriate diagnostic tests and treatments should be made available. It is of note that the prevalences of infections were low in the dogs kept as companion animals and which were reported to seldom have ectoparasites. In contrast, ectoparasites were readily found on dogs from commercial dog breeding facilities and vector-borne agents were identified within these parasites. Veterinarians responsible for animals in such facilities should encourage tick control to prevent unnecessary morbidity and mortality. It should also be borne in mind that the prevalence and importance of vectors may vary considerably due to the influence of climactic and other environmental factors. China is a large country that can be divided into seven geographical regions. We studied dogs from each of these areas and found at least one vector-borne agent in five of the seven geographical regions. Future studies with more comprehensive and representative sampling should be performed to investigate the influence of climactic and environmental impact on the distributions of vectors and their agents in the different regions of China.

*Hepatozoon canis* was the most common vector-borne agent we identified and also the most widespread, being identified in dogs from 5 of the 10 provinces we studied. The organism also occurs widely around the world and, although moderate to severe disease can occur [26], most infections are sub-clinical.

The short (177 bp) and long (591 bp) 18S rRNA nucleotide sequences that we obtained for the *Theileria* sp. we identified were identical to that of *T. orientalis* from India (KM609973) and Japan (XR_696404). *Theileria orientalis* is normally found in yaks (*Bos grunniens*), cattle and buffaloes (*Bos bubalis*) and is transmitted by *Haemaphysalis* spp. [27, 28]. To the best of our knowledge, ours is also the first report that *T. orientalis* or a closely related organism might occur in dogs. How dogs become exposed to this organism in China and the pathogenicity and significance of infections requires further investigation.

Although Wang and Zhang et al. were unsuccessful in identifying *E. canis* infections in dogs in China using serology and PCR [29, 30], other workers have found serological and molecular evidence of infections in the blood of 2 % of dogs in Shenzhen [7–9] and the organism in *R. sanguineus* senso lato ticks from dogs in China [31]. We have now found PCR evidence that *E. canis* infections are relatively common in Taixing, Jiangsu province. Canine ehrlichiosis is a common disease of dogs around the world that is a cause of considerable morbidity and mortality [32]. There are no vaccines available and treatment can be problematic. The disease is best combated by preventing infections with appropriate tick control strategies.

Our findings of *B. vogeli* and *B. gibsoni* at relatively low levels in dogs from Taixing and Nanjing, Jiangsu province, is consistent with a previous report that levels of infection with *Babesia* spp. are low in domestic dogs in China [24]. The relatively low percentage (3.4 %) of ticks we found with *B. vogeli* in Taixing, Jiangsu province, was similar to that reported in other provinces, mainly Guangdong (3.6 %; 1/28), Hainan (3.3 %; 4/121) and Zhejiang (6.7 %; 1/15) [16]. It was, however, lower than that reported in Chongqing (25.0 %; 4/16) and Guangxi (12.5 %; 11/88), indicating there is considerable regional variation in infection rates in China. While *B. vogeli* infections can cause severe disease in puppies, greyhounds and immune-suppressed dogs, infections usually only result in mild signs or are subclinical [4, 33, 34], *B. gibsoni* on the other hand is generally regarded as being more pathogenic and can cause severe disease which responds poorly to drug therapy [35]. There are no vaccines and prevention depends on adequate tick control and, as *B. gibsoni* can be transmitted in blood and by bites during dog fights [4].

We were unable to identify *Anaplasma* spp., *Dirofilaria* spp. and *Leishmania* spp. in our study. This is in contrast to earlier reports from China where *A. phagocytophilum* was demonstrated by PCR in dog ticks from Suifenhe, Heilongjiang province (5.9 %), and in dog blood samples from nine provinces of China (10.9 %; 11/102) [36, 37]. Further, *D. immitis* infections have been demonstrated by microscopic examination and PCR test in dogs from Dandong, Liaoning province (24.0 %; 147/886), and *Leishmania* spp. demonstrated by real-time PCR test in dogs (24.8 %; 78/314) in Sichuan province [12, 13, 36, 37]. While the different observations might have been due to regional differences in infection rates, it is most likely they were due to demographical differences with the dogs in the above studies being more free-ranging and less well cared for and hence more likely to be exposed to ectoparasites. Larger and more inclusive studies are indicated to more accurately determine the prevalences and distribution of vector-borne agents in dogs in China.

## Conclusions

In summary, we found the DNAs of *Babesia gibsoni* and *B. vogeli* (1.2 %), *Ehrlichia canis* (1.3 %), *Hepatozoon canis* (1.8 %), and *Theileria orientalis* (0.1 %) in the bloods of the dogs studied. Further, we found *Babesia vogeli* (3.4 %) and *Ehrlichia canis* (4.1 %) in the *R. sanguineus* senso lato. Our data from 10 provinces in China show a wide range of important vector-borne pathogens occur in dogs and further larger scale studies are indicated to determine more accurate prevalence data for these agents.
